# Influence of Alloy Atoms on Substitution Properties of Hydrogen by Helium in ZrCoH_3_

**DOI:** 10.3390/ma14216704

**Published:** 2021-11-07

**Authors:** Panpan Wang, Qilong Cao, Yuwei You, Xiangshan Kong, Xuebang Wu, Changsong Liu

**Affiliations:** 1 Key Laboratory of Computational Physics of Sichuan Province, Yibin University, Yibin 644000, China; 1982cql@163.com (P.W.); qlcao@mail.ustc.edu.cn (Q.C.); 2 School of Mathematics and Physics, Anhui Jianzhu University, Hefei 230601, China; 3 Key Laboratory for Liquid-Solid Structural Evolution and Processing of Materials (Ministry of Education), Shandong University, Jinan 250061, China; xskong@sdu.edu.cn; 4 Key Laboratory of Materials Physics, Institute of Solid State Physics, Chinese Academy of Sciences, P.O. Box 1129, Hefei 230031, China; xbwu@issp.ac.cn (X.W.); csliu@issp.ac.cn (C.L.)

**Keywords:** ZrCo alloys, disproportionation reaction, fixing helium, ab initio calculations

## Abstract

Intermetallic alloy ZrCo is a good material for storing tritium (T). However, ZrCo is prone to disproportionation reactions during the process of charging and discharging T. Alloying atoms are often added in ZrCo, occupying the Zr or Co site, in order to restrain disproportionation reactions. Meanwhile, T often decays into helium (He), and the purity of T seriously decreases once He escapes from ZrCo. Therefore, it is necessary to understand the influence of alloying atoms on the basic stability property of He. In this work, we perform systematical ab initio calculations to study the stability property of He in ZrCoH_3_ (ZrCo adsorbs the H isotope, forming ZrCoH_3_). The results suggest that the He atom will undergo displacements of 0.31 and 0.12 Å when it substitutes for Co and Zr, respectively. In contrast, the displacements are very large, at 0.67–1.09 Å, for He replacing H. Then, we introduce more than 20 alloying atoms in ZrCo to replace Co and Zr in order to examine the influence of alloying atoms on the stability of He at H sites. It is found that Ti, V, Cr, Mn, Fe, Zn, Nb, Mo, Tc, Ru, Ta, W, Re, and Os replacing Co can increase the substitution energy of H by the He closest to the alloying atom, whereas only Cr, Mn, Fe, Mo, Tc, Ru, Ta, W, Re, and Os replacing Co can increase the substitution energy of H by the He next closest to the alloying atom. The influence of the alloying atom substituting Zr site on the substitution energies is inconspicuous, and only Nb, Mo, Ru, Ta, and W increase the substitution energies of H by the He closest to the alloying atom. The increase in the substitution energy may suggest that these alloy atoms are conducive to fix the He atom in ZrCo and avoid the reduction in tritium purity.

## 1. Introduction

Gaseous tritium (T) is an important fusion raw material in fusion reactors, and is often saved in metallic uranium, which possesses good properties of charging and releasing T [1,2,3,4]. However, uranium has many defects as well, such as spontaneous combustion after the storage of T, being radioactive, and being easy to pulverize. Intermetallic alloy ZrCo is a good substitute [5,6,7,8,9,10,11]. ZrCo has a lower equilibrium pressure of T absorption and a high T absorption [12,13,14]. Compared with uranium, ZrCo is not easy to spontaneously combust, is nonradioactive, and is safe to operate. Meanwhile, ZrCo has the better ability in fixing helium (He) compared with metallic uranium. Although the ZrCo alloy has many advantages in storing T, it also has some defects. ZrCo is transformed to hydride ZrCoT_3_, which decomposes and releases T atoms. However, ZrCo is prone to disproportionate after multiple cycles of absorption and release of T by generating ZrCo_2_ and ZrT_2_ [15,16,17], which are very difficult to decompose: 2ZrCo + T_2_ → ZrCo_2_ + ZrT_2_. The disproportionation reaction of ZrCo eventually results in a decrease in T absorption and the release capacity of ZrCo, and ultimately affects the T storage performance and service life of the alloy. Thus, alloying atoms, such as Ni, Fe, Hf, Sc, Ti, and Mn, are usually used to replace the Zr or Co atom in ZrCo to restrain the disproportionation reaction [18,19,20,21,22]. Extensive experiments have also confirmed that alloying element Ni substituting for Co can inhibit the disproportionation reaction [9,14,18,23,24,25]. It was experimentally found that Ni substituting for Co can prevent the disproportionation reaction and increase the plateau pressure of ZrCo for releasing H at 583 K [18]. Zhang et al. and Jat et al. found that Fe can improve the durability of the anti-disproportionation reaction of ZrCo by replacing the Co atom [19,20]. Peng et al. found that Hf replacing Zr can also restrain the disproportionation rate of ZrCo, where the content of Hf increases from 0 to 30% [26]. Konishi et al. found the similar phenomenon that Hf can avoid the disproportionation reaction of ZrCo by replacing Zr, and ZrCo can reach an ideal plateau pressure for releasing H at a relatively low temperature [27,28]. Another alloying element Ti replacing Zr in the ZrCo alloy can also present an anti-disproportionation effect [19,29,30,31]. Through extensive consideration of the effects of various alloying elements on the disproportionation of ZrCo, Zhao et al. and Qi et al. experimentally found that only Ti substituting for Zr presents an effective anti-disproportionation effect, whereas other alloying elements, such as Sc, Fe, and Ni, instead increase the disproportionation rate in ZrCo [30,31].

Moreover, many theoretical works are performed to study the roles of alloying elements on the disproportionation of ZrCo. Yang et al. systematically investigated the influence of many alloying elements, such as Ti, Y, V, Nb, Ta, Cr, Mn, Ru, Rh, Pd, Zn, and Ni, on the anti-disproportionation ability of the ZrCo alloy, and they confirmed the ability of Ti, Nb, Ta, and V replacing Zr on the anti-disproportionation ability of ZrCo [13,14]. In our previous work, the effects of almost all transition alloying elements replacing Zr and Co on the anti-disproportionation of ZrCo were considered, and the results show that only Ni substituting for Co, and Fe, Co, Ni, Ru, Pd, Os, and Ir replacing Zr exhibited anti-disproportionation roles [32]. In the storage process of T in the form of hydride, many T atoms spontaneously decay into ^3^He atoms, since the half life of T is approximately 12.3 years. There must be many He atoms existing in hydride ZrCoT_3_, and the number of He atoms will gradually increase over time [33]. The accumulation of He inside the defects, such as vacancies and voids, will result in the formation of a He bubble, and thus degrade the T-storage properties of ZrCo. Due to the low electron density around the vacancies, He prefers to situate inside the intrinsic vacancies and form vacancy–helium complexes (V*_n_*He*_m_*), which are the precursors of the bubbles [34,35]. V*_n_*He*_m_* grows by the gain of the chemical potential [36]. These He-driven extended defects agglomerate by the Ostwald ripening process. When the He-induced pressure inside the agglomerated defects (e.g., microcracks) is sufficiently large enough to cause the macroscopic propagation of cracks in the damaged region, lift-off of the top layer occurs in the form of bubbles and/or craters due to the elastic relaxation of cracks [36]. He atoms will reduce the purity of tritium once He bubbles burst and He atoms enter tritium. Hayashi et al. have investigated the long-term release behavior of He from ZrCo and found that the release fractions of He from ZrCo increased to approximately 25% [37]. Therefore, fixing He to avoid its migration and accumulation in ZrCo is also very important. Wang et al. theoretically studied the influence of Ti on the dissolution and migration of He in ZrCo, and found that the presence of Ti increases the migration barrier of He, and thus decreases the diffusion of He [38]. However, there is still a lack of systematic research on the influence of alloying elements added in ZrCo on fixing the He atom. In the present work, systematical ab initio calculations are performed to study the influence of transition alloying elements on the substitution energy of He replacing H in order to access the ability of the fixing He atom in ZrCoH_3_. Co and Zr in ZrCoH_3_ are, respectively, replaced by transition alloying elements. The influence of the alloying atoms on the substitution energies for their closest and next closest H replaced by He is then considered. In the present work, we do not considered the influence of vacancy.

## 2. Computational Details

The present density functional theory calculations are carried out using Vienna Ab initio Simulation Package (VASP) [39,40]. The projector augment approximation wave potentials, the generalized gradient approximation, and the exchange–correlation functional PBE are employed in the calculations [41]. The wave functions are expanded in the plane-wave basis set, and an energy cut-off value of 500 eV is used for ZrCoH_3_ systems. The spin polarization is turned on in all of our calculations. The meshes of 7 × 5 × 7 k-points are used to integrate over the Brillouin zones in the 80-atom ZrCoH_3_ systems (containing 48 H, 16 Co, and 16 Zr atoms), respectively. We fully optimized the shape, volume, and atom position in the ZrCoH_3_ system. Once the forces on each atom in the supercell are less than 0.01 eV/Å, the optimization calculations will be terminated. The stability of He is determined by formation energies and substitution energies, which are calculated by the expression:

*E*^*For/Sub*^ = *E*^*X*^ − *E^P^* − Σ*n_i_ƞ_i_* (1)

In the expression, *E^X^* and *E^P^* are the total energies of the supercells with and without *X*, respectively. *ƞ_i_* is the chemical potential of impurity species *i*, and *n_i_* is the number of the species *i* added to (*n_i_* is larger than 0) or removed from (*n_i_* is less than 0) the supercells. In calculating the substitution energy of H at 8*e* site by He, we assume that ZrCo is already disproportionate and the 8*e* site is occupied by the He atom.

## 3. Results and Discussion

### 3.1. Basic Lattice Parameters and Vacancy Formation Energies

The space group of ZrCo is *Pm*-*3m* numbered 221, and the unit cell has only one lattice constant *a* = 3.18 Å, which is consistent with previous results [42,43]. When ZrCo is charged with H, it changes to ZrCoH_3_. The symmetry group of ZrCoH_3_ is *Cmcm* numbered 63. The three angles are all 90°, and ZrCoH_3_ has three lattice parameters: *a*, *b*, and *c*. In order to validate the parameters chosen here, we optimize the unit cell of ZrCoH_3_ and obtain the lattice parameters of the ground-state structure. The parameters, together with the available reference values, are summarized in Table 1. From the table, one can see that the calculated parameters *a*, *b*, and *c* are very close to the available reference values published in previous work. The differences are no more than 0.06 Å, which is within the normal margin of error [43,44,45].

Moreover, we also calculate the formation energies of vacancies located at Zr, Co, and H sites. The results are also listed in Table 1. No available reference values can be found for comparison. The results suggest that Zr vacancy formation energy is the largest, at 3.38 eV, followed by the Co vacancy formation energy of 0.97 eV. In contrast, the vacancy formation energies at H sites are relatively small, and the vacancy formation energies are 0.45–0.55 eV. The results suggest that it is most unfavorable to form vacancies at Zr sites, whereas it is most energetically favorable to form vacancies at H sites.

### 3.2. Stability of Helium at 8e, Zr, Co and H Sites

**Figure 1:**
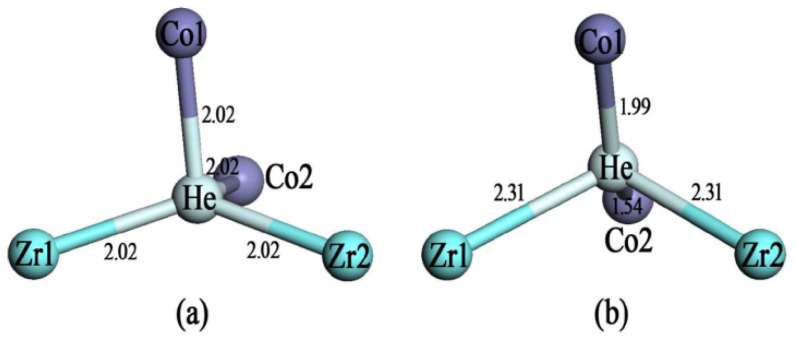
The two 8*e* positions (**a**) and (**b**) occupied by He around the 8*e* site. The distances (Å) among the atoms are also labeled. Zr1 and Zr2 are the labeled two Zr atoms surrounding an 8*e* site, and Co1 and Co2 are the labeled two Co atoms surrounding an 8*e* site. He suggests the positions occupied by the He atom. The bond lengths are also labeled.

It was generally thought that the occupation of hydrogen at 8*e* sites results in the disproportionation reaction in ZrCoH_3_. The 8*e* site refers to the center position in the tetrahedron formed by the two closest Zr atoms and two closest Co atoms. Yang et al. have systematically simulated the occupation of H at the 8*e* site in ZrCoH_3_ and the influence of alloying atoms substituting Zr and Co on the occupation of H at the 8*e* site [44]. Once the hydrogen isotope T spontaneously decays into the ^3^He atom, the stability of He at 8*e* sites will have a great influence on the purity of released T. Therefore, it is of great importance to study the stability of He at 8*e* sites. We consider the occupation of He at the 8*e* sites, and the formation energies of He at these sites are calculated according to Equation (1). From our results, once a He atom is put at the 8*e* site, it will move spontaneously to two occupation sites, as shown in Figure 1a,b. In Figure 1a, the distances of He to both Zr1 and Zr2, and both Co1 and Co2, are the same, at 2.02 Å. At this position, the substitution energy of H by He is 3.62 eV. The distances of He in Figure 1b to both Zr atoms are the same, at 2.31 Å, whereas the distances of He to Co1 and Co2 are only 1.54 Å and 1.99 Å, respectively. The substitution energy of H by He at this situation is relatively large, at 4.06 eV. In the absence of H or He, the distances from the 8*e* site to the two closest Zr atoms are 1.97 Å, and the distances from the 8*e* site to the two closest Co atoms are 1.59 Å. Therefore, it can be seen that the presence of He at the 8*e* site pushes the surrounding Zr and Co atoms away. This can be understood by the unfavorable interaction of closed-shell He with surrounding Co and Zr atoms.

**Figure 2:**
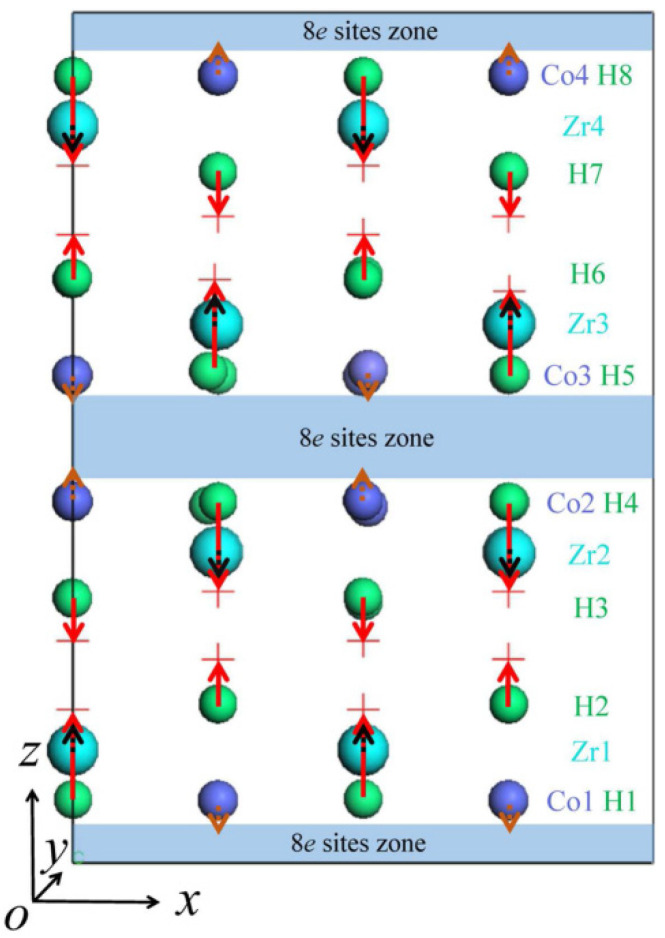
The front view of the supercell used here. The arrows suggest the movement directions, and the length of the arrow indicates the size of the movement. The green, purple, and cyan balls suggest the occupying positions of He at H, Co, and Zr sites, respectively. The crosses are the final positions of He atoms. The letters Co*n*, Zr*n*, and H*n* labeled in the right side are the *n*th line of Co, Zr, and H, respectively. The blue zones are the 8*e* site zones.

When ZrCo adsorbs T and becomes ZrCoT_3_, many T atoms may spontaneously decay into ^3^He atoms at T sites. The movement of He may also substitute Zr or Co atoms. Therefore, we consider the substitution of Zr, Co, and H at lattice point sites by He atoms. According to our calculations, all of the He atoms located at Zr, Co, and H sites will move spontaneously. In order to clearly elucidate the movement of He atoms at Zr, Co, and H sites, we mark the movement directions of He atoms, as shown in Figure 2. We firstly discuss the movement of He atoms located at Co sites. In our established supercell, the first, second, third, and fourth-line Co atoms are marked Co1, Co2, Co3, and Co4, respectively. The movement direction and distance of the He atoms replacing the same line of Co sites are the same. According to our calculations, He atoms will move by 0.31 Å towards 8*e* sites zone once the He atom is put at Co sites at the first-line Co sites. The similar migrations of He atoms towards the 8*e* sites zone by 0.31 Å are found for He atoms at the second, third, and fourth-line Co sites. In contrast, He atoms will migrate away from the 8*e* site zone, as shown in Figure 2, when He atoms are located at Zr sites. The first, second, third, and fourth-line Zr sites are marked Zr1, Zr2, Zr3, and Zr4, respectively. The migration distances of He atoms from Zr sites at the first, second, third, and fourth-line are only 0.12 Å. The largest movement is made by He atoms situated at H sites. He atoms at first-line H sites move away from the 8*e* site zone. The first to eighth-line H sites are marked H1 to H8, respectively. The movement distances of He at H sites are approximately 1.09 Å. The similar movement style is found for He atoms situated at fourth, fifth, and eighth-line H sites. He atoms replacing H atoms at the second-line move away from the 8*e* site zone to the interior interstitial sites, and the movement distance is approximately 0.67 Å. The movements of the He atoms are not directly along the *z*-axis, whereas there is a component along the *y*-axis. The movement of He atoms from the Co, Zr, and H sites is due to the electron structure of He. The He atom is energetically unfavorable to hybridize with surrounding H, Co, and Zr atoms since it is a closed-shell atom. He atoms move away from their initial sites to keep sufficient distance from their neighboring H, Co, and Zr atoms.

In term of the stability of He atoms at the sites considered above, we calculate the substitution energies for H replaced by He. According to our results, the substitution energies of He atoms at Co sites are all 2.86 eV. In contrast, the substitution energies of He at Zr sites are all 4.05 eV. The substitution energies for all Co atoms replaced by He are the same, which is consistent with the same movement distances of He atoms located at Co sites. The similar situation is found for He at all Zr sites. Therefore, He atoms at Co sites are relatively stable. There are two kinds of movement styles of He atoms located at H sites, which, in turn, results in two types of substitution energies of 2.81 eV and 3.40 eV. The formation energies of 2.81 eV and 3.40 eV correspond to the He movements of 0.67 Å and 1.09 Å, respectively. The relatively large substitution energy is due to the closer distance of He to its closest neighbors.

### 3.3. Effects of Alloying Elements at Co Site on Stability of Helium

**Figure 3:**
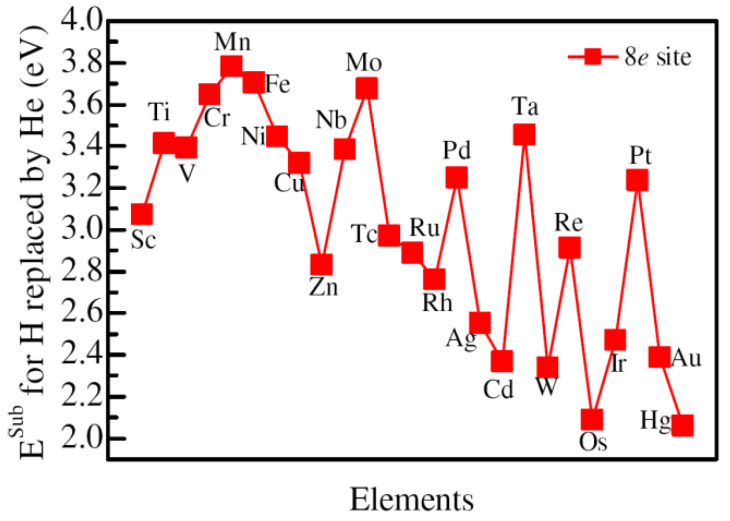
The substitution energy of He replacing H at the 8*e* site with the closest Co atom is substituted by various alloying elements.

As discussed in the introduction section, alloying atoms are often added in ZrCo to restrain the disproportionation reaction. Meanwhile, T atoms often spontaneously decay into ^3^He atoms in the storage process of T in the form of ZrCoT_3_. Therefore, it is necessary to understand the influence of alloying atoms on the stability of He. The possible occupancy sites for alloying atoms in ZrCoT_3_ are either the Zr or Co site. Firstly, we consider the occupancy of the alloying atom at the Co site, that is, the alloying atom substituting for the Co atom. Since all of the Co sites in the supercell are equivalent, we only consider one Co atom replaced by an alloying atom. If the Co atom in the supercell is replaced by an alloying atom, then He atoms situated at its closest 8*e* site, its closest H site, and its next closest H site are considered. The substitution energies are calculated for H at the three sites replaced by He. The results for He at 8*e* sites are presented in Figure 3. One can see that the substitution energy increases from Sc to Mn, and then decreases to Zn for 3*d* alloying elements. The substitution energy for He replacing H at the 8*e* site closest to Mn is the largest, whereas the substitution energy is the smallest for He replacing H at the 8*e* site closest to Zn. For 4*d* alloying elements, there are two peaks for the substitution energy, which correspond to Mo and Pd. The lowest substitution energy is in the situation where He replaces H at the 8*e* site closest to Cd. In contrast, there are three peaks of the substitution energy for 5*d* alloying elements, and the three peaks correspond to Ta, Re, and Pt, respectively. The lowest substitution energy is found for the last element in 5*d*, similar to that of 3*d* and 4*d* alloying elements. In the above section, it is known that the lowest substitution energy of He replacing H at the 8*e* site without adding alloying atoms is 3.62 eV. Therefore, the presence of many kinds of alloying atoms decreases the substitution energy, except the 3*d* elements Cr, Mn, Fe, Ni, and Cu and 4*d* elements Nb, Mo, and Pd, as well as 5*d* element Pt. The increase in the substitution energy suggests that the existence of these alloy atoms may not be conducive to the replacement of H at the 8*e* site by He.

**Figure 4:**
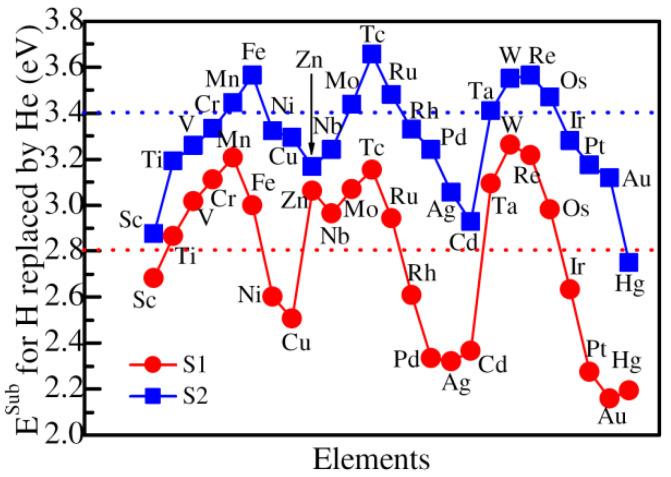
The substitution energy of He replacing H at the closest site of the Co atom, which is substituted by various alloying elements. S1 and S2 suggest the closest and next closest sites to Co substituted by 3*d*, 4*d*, and 5*d* alloying elements.

As for the substitution energies of He replacing H at the closest and next closest sites to Co substituted by 3*d*, 4*d*, and 5*d* alloying elements, the substitution energies are plotted in Figure 4. From the figure, one can see that the substitution energy for He replacing H at the closest site increases from Sc (2.68 eV) to Mn (3.21 eV), and then decreases to Cu (2.51 eV) in 3*d* elements. As for 4*d* elements, the substitution energy increases from Nb (2.96 eV) to Tc (3.15 eV), and then decreases to Ag (2.32 eV). In contrast, the substitution energy increases from Ta (3.09 eV) to W (3.26 eV), and then decreases sharply to Au (2.16 eV). Compared with the closest site, the substitution energies of He replacing H at the next closest site are relatively large. In 3*d*, 4*d*, and 5*d* alloying elements, the largest substitution energies are 3.56 eV, 3.65 eV, and 3.55 eV for Fe, Tc, and Re, respectively. According to the calculated results in the context, the substitution energy of He at the next closest site is 2.86 eV in ZrCoH_3_ without adding any alloying atoms. Therefore, it can be seen that the replacement of Co by the alloying atoms, such as Ti, V, Cr, Mn, Fe, Zn, Nb, Mo, Tc, Ru, Ta, W, Re, and Os, can increase the substitution energy of H by He. The results suggest that the presence of the alloying atoms are conducive to the replacement of the lattice point H by He.

### 3.4. Effects of Alloying Elements Replacing Zr on Stability of Helium

**Figure 5:**
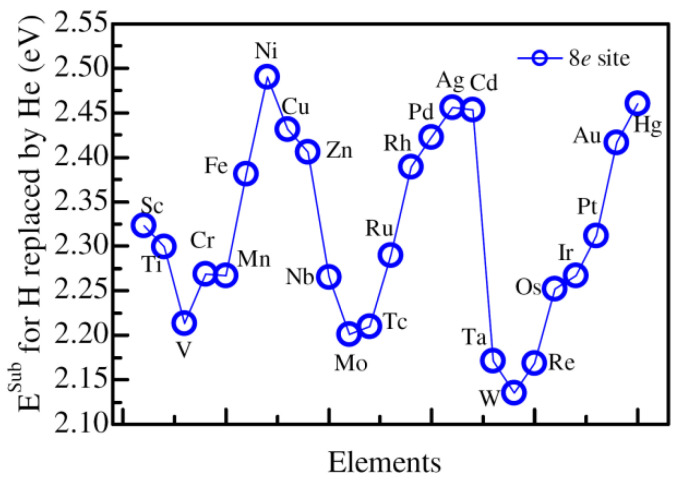
The substitution energy of He replacing H at the 8*e* site closest to the Zr atom, which is substituted by various alloying elements.

Similar to the calculations for the influence of alloying atoms on the substitution energies of H by He, we evaluate the substitution energies for He replacing H at 8*e* sites. The closest and next closest lattice sites to Zr are replaced by 3*d*, 4*d*, and 5*d* alloying elements. Figure 5 shows the substitution energy of He replacing H at the closest 8*e* site of Zr, which is replaced by alloying atoms. According to the obtained results, the substitution energy firstly decreases from Sc (2.32 eV) to V (2.21 eV), and then increases sharply to Ni (2.49 eV). Thus, the substitution energy is the largest for He replacing H at the closest 8*e* site of Zr replaced by Ni in 3*d* alloying elements. For 4*d* alloying elements replacing Zr, the substitution energy for He substituting H closest to Zr decreases from Nb (2.26 eV) to the lowest Mo (2.20 eV), and then increases to Ag (2.46 eV). The similar change tendency is found for Zr substituted by 5*d* alloying elements. The substitution energy for He replacing H closest to W is the lowest (2.14 eV), and the substitution energy for the situation of Zr replaced by Hg is the largest, at 2.46 eV. In general, the substitution energy for He substituting H closest Zr replaced by alloying atoms differs very little, at only 0.35 eV. Compared with the substitution energy for He substituting H closest to Zr, the substitution energy is as high as 3.62 eV. Therefore, the presence of the alloying atom substantially decreases the substitution energy for He replacing H at the 8*e* site closest to the alloying atoms.

**Figure 6:**
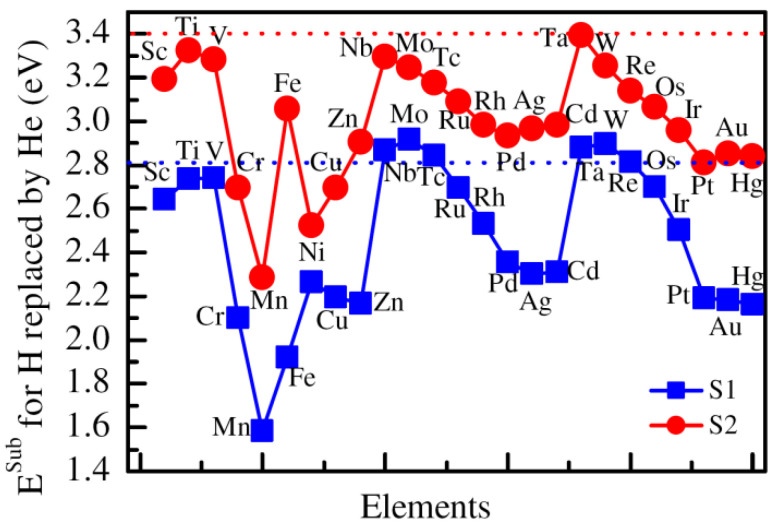
The substitution energy of He replacing H at the closest site of Zr atom, which is substituted by various alloying elements. S1 and S2 suggest the closest and next closest sites to Zr substituted by 3*d*, 4*d*, and 5*d* alloying elements.

Meanwhile, we also consider the substitution energy of He replacing H at lattice point sites closest and next closest to Zr. The Zr atom is replaced by 3*d*, 4*d*, and 5*d* alloying elements, and the calculated substitution energy is plotted in Figure 6. From the figure, it can be seen that the substitution energy of He replacing H at the closest site of Zr has the largest value when the Zr atom is substituted by Ti and V, and the smallest value when the Zr atom is replaced by Mn in 3*d* alloying elements. The substitution energy of He replacing H at the closest site of Zr is the largest once Zr is substituted by Ni in 4*d* alloying elements, and the smallest value is found for He replacing H when Zr is replaced by Ag. The largest and smallest substitution energies for H replaced by He are found when Zr is substituted by 5*d* alloying elements W and Hg, respectively. For all alloying elements, the largest and smallest substitution energies are found for H substituted by He at the closest site of Zr replaced by Mo and Mn, respectively. In contrast, the largest and smallest substitution energies for H at the next closest site substituted by He are found when Zr is replaced by Ti and Mn, respectively. Compared with the substitution energy of H by He (2.86 eV) close to Zr, the substitution energies of H at the closest site of Zr are generally reduced, whereas the substitution energies of H at the next closest site of Zr are mostly enlarged.

## 4. Discussion

**Figure 7:**
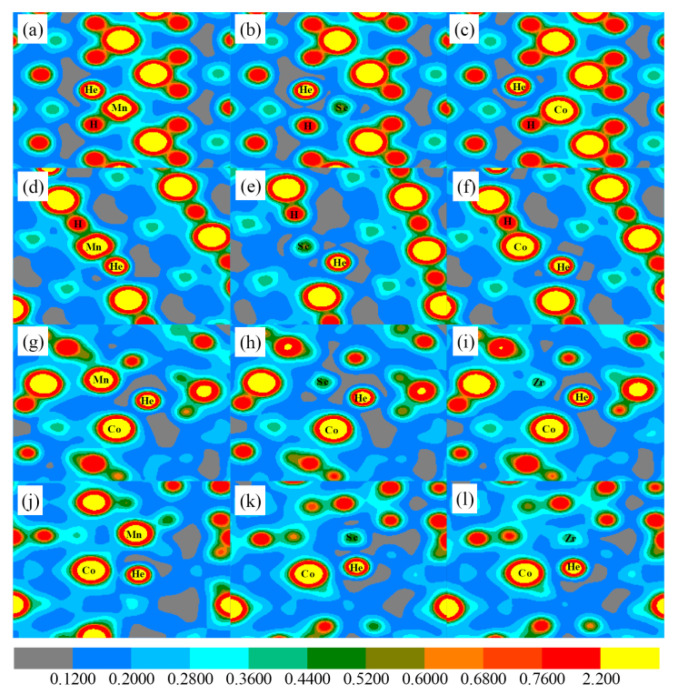
(**a**–**c**) The charge density map across H, the closest He of Mn, Sc, and Co at Co site, and Mn, Sc, and Co, respectively. (**d**–**f**) The charge density map across H, the next closest He of Mn, Sc, and Co at Co site, and Mn, Sc, and Co, respectively. (**g**–**i**) The charge density map across H, the closest He of Mn, Sc, and Zr at Zr site, and Mn, Sc, and Zr, respectively. (**j**–**l**) The charge density map across H, the next closest He of Mn, Sc, and Zr at Zr site, and Mn, Sc, and Zr, respectively.

The underlying reasons controlling the influence of alloying atoms on the substitution of H by He atoms are discussed. We calculate the charge density map for He substituting the lattice H atoms. The situations of He closest and next closest to Co and Zr are considered. The Co and Zr are replaced by alloying atoms (we take alloying atoms Mn and Sc as an example, as shown in Figure 7). Figure 7 a,b are the situations where Co are substituted by Mn and Sc, respectively. Figure 7c is the situation where Co is not replaced by any alloying atom, and where He atoms replace the closest H atom of Mn, Sc, and Co. From the figure, one can see that the charge density between Mn and H is relatively high, suggesting hybridization. In contrast, the charge density between Mn and He is lower. The similar situation is found for Co without being replaced by any other alloying atom. As for Sc replacing the Co atom, the charge density between Sc and He (as well as H) is very low. Figure 7d–f are still the situations of lattice Co atoms replaced by Mn, Sc, and Co, respectively. The difference is that He substitutes the next closest H atom of Co. In the same way, the charge density between the alloying atom and He is very low, whereas the charge density between Mn (Co) and H is relatively high. The charge density around He is very low. Similar situations are found for Zr (its closest and next closest H is replaced by He) replaced by Mn and Sc. The reason why He is preferable for occupying the sites with a very low charge density nearby is that He is a closed-shell atom. Due to the fact that helium is a closed-shell atom, it will be chemically unfavorable to bind with neighboring H, Co, Zr, and alloying atoms, which will inevitably result in local structure distortion and will affect the storage and release of T. In contrast, H is an unfilled-shell atom, and is energetically favorable to bind with surrounding Co, Zr, and alloying atoms. With the increase in He in ZrCo, the local lattice is often distorted and free space is enlarged. The enlarged free space, in turn, provides more sites for He to accumulate in ZrCo. Once the stability of He is low, the He atom may escape from the lattice site and move into ZrCo, which provide conditions for He to accumulate in ZrCo. The accumulation of He inside ZrCo will result in the formation of a He bubble, and thus degrade the H storage properties of ZrCo. Above all, the purity of H is substantially reduced once the He atoms migrate out of ZrCoH_3_ through the bursting of the He bubble. Therefore, the high stability of He in ZrCo is of great concern.

By carrying out systemical ab inito calculations, we determined the stability of He in ZrCo and the influence of alloying atoms on the stability of He in ZrCo. It was found that the alloying atoms Ti, V, Cr, Mn, Fe, Zn, Nb, Mo, Tc, Ru, Ta, W, Re, and Os replacing Co can increase the substitution energy of H by He at the closest site of alloying atoms (that is, the H site that belongs to H5 in Figure 2). Meanwhile, only the alloying atoms Cr, Mn, Fe, Mo, Tc, Ru, Ta, W, Re, and Os replacing Co can increase the substitution energy of H by He at the next closest site of alloying atoms (that is, the H site that belongs to H6 in Figure 2). In contrast, the influence of the alloying atom substituting Zr site on the substitution energies of H at the closest site of alloying atoms and the next closest site of alloying atoms by He is inconspicuous. All of the substitution energies of H by He at the next closest site of alloying atoms are reduced by alloying atoms at the Zr site, and only Nb, Mo, Ru, Ta, and W increase the substitution energies of H by He at the closest site of alloying atoms. The increase in the substitution energy may suggest that the existence of these alloy atoms is conducive to fix the He atom in ZrCoH_3_. In this way, the He atom is difficult to move, and accumulates to form a bubble and reduce the purity of H.

## 5. Conclusions

In the present work, we perform systematical ab initio calculations to study the substitution property of H by He in ZrCoH_3_, and the influence of more than 20 alloying atoms replacing the Co and Zr site on the substitution energy of He at H sites. The results suggest that the He atom replacing Co, Zr, or H will undergo serious displacements. He displaces 0.31 and 0.12 Å when it substitutes Co and Zr, respectively. In contrast, the displacements are very large, at 0.67–1.09 Å for He replacing H. It is found that alloying atoms Ti, V, Cr, Mn, Fe, Zn, Nb, Mo, Tc, Ru, Ta, W, Re, and Os replacing Co can increase the substitution energy of H by the He closest to the alloying atom, whereas only Cr, Mn, Fe, Mo, Tc, Ru, Ta, W, Re, and Os replacing Co can increase the substitution energy of H by the He next closest to the alloying atom. In contrast, the influence of the alloying atom substituting Zr site on the substitution energies is inconspicuous, and only Nb, Mo, Ru, Ta, and W increase the substitution energies of H by the He closest to the alloying atom. The increase in the substitution energy may suggest that these alloy atoms are conducive to fix the He atom in ZrCo and reduce He release.

## Figures and Tables

**Table 1:** The lattice parameters, as well as vacancy formation energies, in ZrCoH_3_. The available reference values are also listed here for comparison.

Object	Present	Reference
*Latt*(*a*) (Å)	3.54	3.51–3.54 [43,44,45]
*Latt*(*b*) (Å)	10.41	10.40–10.48 [43,44,45]
*Latt*(*c*) (Å)	4.32	4.30–4.33 [43,44,45]
*E^ZrVac^_f_* (eV)	3.38	-
*E^CoVac^_f_* (eV)	0.97	-
*E^HVac1^_f_* (eV)	0.55	-
*E^HVac2^_f_* (eV)	0.45	-
